# The Destruction of Bugs and Other Vermin

**Published:** 1910-01-29

**Authors:** 


### THE DESTRUCTION OF BUGS AND OTHER VERMIN.

### THE HYDROCYANIC-ACID-GAS METHOD.

The accidental contamination of a bedstead, a dwelling-room, or of upholstered furniture by bugs or other vermin may occur even in the houses of the well-to-do, and among the poorer classes the difficulty of destroying these vermin is often regarded as so great that families are apt to resign themselves to the presence of these pests in their dwelling-rooms. It may often be of service, therefore, to know a satisfactory method for cleansing houses without the necessity of destroying the furniture, putting the individuals to any great expense, and, when proper precautions are taken, without danger to the inhabitants, provided they are able to vacate for a day or two the rooms that are to be treated. Mr. Fletcher Barrett, of the Incorporated Society for the Destruction of Vermin, has given a detailed report of experiments undertaken on behalf of the Society for the destruction of vermin, and pointed out that entomologists have long noticed that insects vary greatly in their susceptibility to cyanide fumes. The experience gained, however, indicates that the use of hydrocyanic-acid-gas in houses is successful against cockroaches, bedbugs, fleas, clothes moths, ants, white ants, house flies, and other soft-bodied insects; and, as these constitute the majority of the household pests, the use of the gas must now be considered a standard remedy. Moreover, rats and mice are also killed by its use, and it fortunately has the effect of first causing these animals to rush out from their holes into the open, so that the subsequent annoyance of dead mice in walls and under floorings is not experienced. The hydrocyanic-acid-gas is generated from potassium cyanide by means of sulphuric acid, and the general directions for treatment may be briefly summarised as follows:—

(1) Prepare a tabular statement showing room capacity and amount of chemicals for each compartment. (2) Arrange for opening of doors and windows from the outside at the conclusion of the fumigation, and close all registers, fireplaces, and other openings. Do the necessary caulking and remove carpets, rugs, moist-food material, and any metallic objects which are likely to be affected by the fumes. (3) Place the generating vessels in each room with a thick layer of old newspapers under each. (4) Break up the cyanide out of doors and place it in thin paper bags containing a pound or a half-pound each, according to the amounts to be used in the different rooms. (5) Measure into each of the generating jars the proper amount of water, and afterwards add the acid slowly in the proper amount of each of the jars. (6) Place the cyanide in the bags alongside the generating jars in each room. (7) Start at the top of the house and place the cyanide gently into each jar, and quickly leave the room. As soon as the upper floor is finished proceed to the next lower, and pass in this manner from floor to floor until the basement is reached, and exit is made through the lower door. If two persons work together in this operation, they should both be on the same floor together, taking different rooms. (8) On the following day open the windows and doors from the outside, and let the house ventilate for an hour before entering it. (9) After the house is thoroughly ventilated and the odour of the gas has disappeared, the jars should be emptied through the sewer-trap and thoroughly washed.

In order to destroy household insects 1 fl. oz. of commercial sulphuric acid, diluted with 2 oz. of water, and 1 oz. of potassium cyanide (95 per cent.) must be used for every 100 cubic feet of space. For loosely constructed framehouses these amounts should be doubled per 100 cubic feet.

The experiments were made upon a well-built, semi-detached villa, containing eleven rooms. The house was vacated and all liquid and solid foods were removed. The cubic content of each room was carefuly computed, and a tabular statement prepared. All the windows were closed, and, where necessary, sealed with moist paper, and the doors and windows were left unlocked, so that they could be opened from the outside. The fireplace flues were stuffed with paper and the registers closed.

Two-gallon earthenware jars were used as generating vessels, one or more being placed in each room; under each jar a thick layer of old newspaper was placed. When everything was made ready the requisite amount of water was poured into the jars, the proper amount of sulphuric acid added slowly, and the potassium cyanide in the packets was gently placed into the vessels prepared to receive them. Having finished the top floor, the operators passed rapidly to the next, and so on to the basement, making their exit through the lower door into the garden. The house was then locked up from the outside and notices put up to caution against entrance.

Next morning the doors and windows were opened from the outside, and, after allowing some time to elapse in order that the house might be aired, it was entered and all the windows opened, and kept open for several hours. All water-taps throughout the house were set running until the cisterns were emptied, lest the water might have absorbed any of the poisonous gas, and the contents of the generating jars were poured directly down the sewers, and the jars at once thoroughly cleansed.

The work of fumigation should be placed in the hands of a trained person, who would carefully consider all the recommendations and precautions and become thoroughly familiarised with them before undertaking the experiment. It follows from what has been said that there may be danger from fumigating one house in a row of houses separated only by party-walls, the other house being inhabited. Unnoticed cracks in a wall would admit the poisonous gas to the neighbouring houses. In such a case a householder must consult his neighbours.

---

At the annual meeting of the Leeds Hospital for Women and Children on January 20, it was proposed in conjunction with the general infirmary and the dispensary, to appoint an almoner for six months as an experiment, to see how much work could be organised, and to secure that the charities should be used by the right people.

